# Oxygen-Driven Stabilization and Electrostatic Asymmetry in Janus Mo- and W‑Based Transition Metal Dichalcogenide Nanotriangular Quantum Dots

**DOI:** 10.1021/acsomega.6c00678

**Published:** 2026-04-30

**Authors:** Jair Othoniel Dominguez Godinez, Raul Eduardo Santoy Flores, José Israel Paez Ornelas, Rodrigo Ponce Pérez, Luis Pellegrin, Do Minh Hoat, Jonathan Guerrero Sánchez

**Affiliations:** † 7082 Centro de Investigación Científica y de Educación Superior de Ensenada, Ensenada, Baja California 22860, México; ‡ Universidad Nacional Autónoma de México, 87793 Centro de Nanociencias y Nanotecnología, Ensenada, Baja California 22860, México; § Universidad Autónoma de Baja California, 27758 Facultad de Ciencias, Ensenada, Baja California 22860, México; ∥ Institute of Theoretical and Applied Research, Duy Tan University, Ha Noi 100000, Vietnam; ⊥ 374802 School of Engineering and Technology, Duy Tan University, Da Nang 550000, Vietnam

## Abstract

Janus transition-metal dichalcogenide nanotriangular quantum dots provide a unique platform for engineering internal electrostatic asymmetry at the nanoscale. In this manuscript, we present a systematic investigation of pristine, nonoxygen Janus, and oxygen-containing Janus Mo- and W-based nanotriangles, in which size, edge geometry, and the chemical composition of the chalcogens govern their stability, curvature, and electrostatic response. The oxygen-containing counterparts are the most stable, followed by the nonoxygen Janus and finally, the pristine nanotriangles. Edge configuration is favored due to reinforced metal–oxygen bonding, as confirmed by ab initio molecular dynamics, which showed stability up to room temperature. The electrostatic potential analysis revealed pronounced potential gradients and spatially separated charge accumulation in oxygen-containing systems, with enhanced curvature and internal polarization. The nonoxygen-containing Janus nanotriangles also produce curvature, but less pronounced because of the smaller electronegativity difference. These effects are systematically stronger in W-based nanotriangles. Oxygen incorporation, edge geometry, and chalcogen identity serve as key descriptors governing the stability and internal electrostatic fields of Janus transition-metal dichalcogenide nanotriangles. The results presented here establish the foundation for a rational design of quantum dots useful in photocatalysis applications.

## Graphical abstract

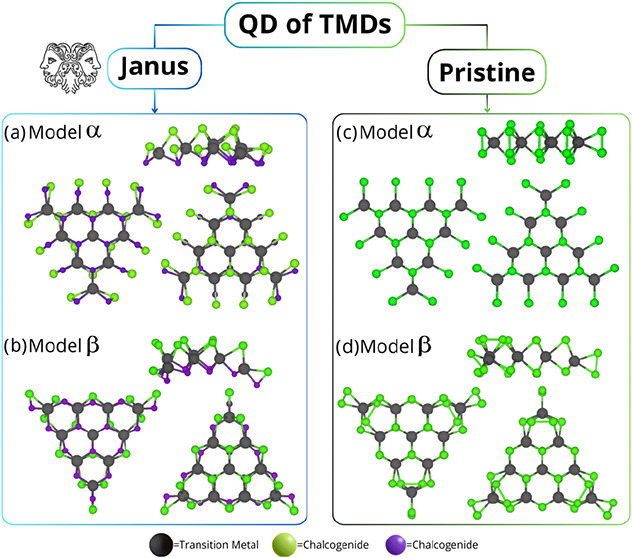

## 1 Introduction

Quantum dots (QDs) are nanoscale semiconductor particles that exhibit size-dependent optical and electronic properties due to quantum confinement effects.^1,2^ Their dimensions, typically ranging from 2 to 10 nm, result in discrete energy levels and tunable band gaps, not present in their bulk counterparts. These unique quantum effects have positioned QDs as fundamental materials for various technological applications, particularly in optoelectronics, quantum computing, photovoltaics, biomedical imaging, and catalysis.^3–6^ The importance of QDs has been recognized globally in the pioneering work about the synthesis of quantum dots.^7^ Their work demonstrated how QDs could be precisely engineered to exhibit tunable electronic and optical properties, revolutionizing applications in nanotechnology. QDs are currently utilized in high-efficiency light-emitting diodes (LEDs), next-generation displays, quantum computing devices, and even biolabeling for medical diagnostics.^4,7^

Janus nanoparticles are a distinct class of nanomaterials characterized by two chemically different surfaces, resulting in asymmetric charge density and an intrinsic dipole.^8^ They have garnered significant interest in nanoscience due to their ability to achieve selective functionalization, enhance reactivity, and exhibit directional interactions. Janus nanomaterials have demonstrated promising applications in optical imaging, smart materials, and interfacial chemistry.^9^ They also show an anisotropic behavior, which is highly versatile for applications in water remediation^10^ catalysis, drug delivery, and self-assembly processes.^11^

Focusing on an important family of nanomaterials, the transition metal dichalcogenides (TMDs) have the chemical formula *MX*_2_, where *M* is a transition metal and *X* is a chalcogen.^12^ These materials can form various nanostructures, including quantum dots with triangular geometry,^13^ nanotubes,^14^ and two-dimensional (2D) monolayers.^15^ The TMDs exhibit a wide range of electronic and catalytic properties, making them highly relevant for applications such as field-effect transistors (FETs),^16^ spintronics,^17^ photovoltaics,^18^ and catalysis, particularly in the hydrogen evolution reaction (HER).^19^ Janus TMDs are formed by selectively replacing one layer of chalcogen atoms in the pristine TMD nanostructure, resulting in a chemical formula *MXY*, where *Y* is a chalcogen different from *X*.^20^ Such structural modification introduces charge-density asymmetry, breaking inversion symmetry and creating unique and tunable properties.^21^ The band gap is an evident change in properties from pristine to Janus monolayers. Pristine *MoS*_2_ and *MoSe*_2_ exhibit direct band gaps of 1.67 eV^22^ and 1.58 eV,^23^ respectively, while their Janus counterparts, *MoSO* and *MoSeO*, show indirect band gaps of 1.07 eV^22^ and 0.81 eV,^24^ that is not it, there is still room for understandig their oxidation patterns, the coverage at which the band gap transition happens, and their further applications.

The study of triangular TMD quantum dots (QDs) is particularly relevant due to their potential catalytic applications.^25^ Among them, *MoS*_2_ has been widely investigated both theoretically^26^ and experimentally.^13^ The morphology and electronic structure of *MoS*_2_ nanocrystals strongly depend on their size.^27^ These triangular QDs can be synthesized via epitaxial growth on Au(111) surfaces.^13^ The edges and vertices of these nanostructures play a crucial role in catalysis. Previous studies have shown that the most stable terminations of *MoS*_2_ QDs are sulfur-rich, leading to one-dimensional metallic edge states.^28^ These edge states significantly enhance catalytic activity, particularly in hydrodesulfurization (HDS) reactions.^29–31^ The ability of these QDs to adsorb and react with sulfur-containing molecules has been studied extensively, including the work of Tuxen et al.,^32^ which demonstrated that *MoS*_2_ QDs with fewer than six *Mo* atoms at the vertices exhibit strong adsorption of dibenzothiophene (DBT).

Building on these findings, we systematically investigate Janus TMD QDs using first-principles calculations. Specifically, we explore two geometrical models (α and β) reported as the most stable structures for Janus MoSeS QDs in ref 33. We systematically analyze their structure and electronic properties by varying their size (*n* = 4 to 10 transition metal atoms along the edge). Our study includes: (1) a comparison between oxidized (*MXO*), nonoxidized (*MXY*), and pristine (*MX*_2_) QDs; (2) an analysis of charge-density asymmetry through electrostatic potential surfaces; (3) the evaluation of surface formation energies to assess chemical stability; (4) a discussion on the thermodynamic stability through ab initio molecular dynamics of these materials.

This article is structured as follows: Section 2 presents the computational details, Section 3 discusses the optimized QDs, their thermodynamic stability, and characterizes their structure and electronic properties, and finally, Section 4 provides conclusions, potential applications, and future directions.

## 2 Computational Details

To investigate the properties of TMD quantum dots (QDs), we employed first-principles calculations within the density functional theory (DFT) framework. All simulations were performed using the Vienna Ab Initio Simulation Package (VASP).^34–36^ A simulation cell with dimensions of *x* = 24 Å,, *y* = 24 Å,, and *z* = 16 Å, was used to create vacuum space between periodic images, which was increased proportionally with the QD size to prevent interactions between replicas. The electronic states were expanded in plane waves with a cutoff energy of 500 eV for all models. The valence states were treated using the frozen-core approximation within the projected augmented wave (PAW) method.^37^ Exchange–correlation interactions were described using the generalized gradient approximation (GGA) with the Perdew–Burke–Ernzerhof (PBE) parametrization.^38,39^ Structural optimization was carried out until the forces on each atom were below 0.01 eV/Å, and the total energy change was less than 1 × 10^–4^ eV. The Brillouin zone was sampled using the γ point. To compute the chemical potentials of each atomic species, we first considered an *O*_2_ molecule in a cubic box of 15 Å, with a *k*-point sampling of 1 × 1 × 1. For sulfur, we used the bulk phase with a primitive cell of triclinic geometry and lattice parameters of *a* = 14.08 Å, *b* = 13.56 Å, and *c* = 8.49 Å, with a Brillouin zone sampling of 6 × 6 × 4. For selenium, we considered a monoclinic conventional cell with lattice parameters of *a* = 9.54 Å, *b* = 14.93 Å, and *c* = 15.47 Å, and a Brillouin zone sampling of 6 × 4 × 3. Finally, for tellurium, we employed a trigonal conventional cell with lattice parameters of *a* = *b* = 4.56 Å, and *c* = 5.90 Å,, with a Brillouin zone sampling of 6 × 6 × 12. For transition metals (*Mo* and *W*), a cubic symmetry was assumed, using a *k*-point grid of 5 × 5 × 5 and lattice parameters of *a* = 3.10 Å, and *a* = 3.19 Å, respectively. These bulk structures are the ones known as most stable in nature. The bulk phases of pristine TMDs 2*H*–*MX*_2_ (where *M* = *Mo*, *W* and *X* = *S*, *Se*, *Te*) were modeled using a *k*-point sampling of 10 × 10 × 5, in agreement with established databases.^40^ To assess the thermal stability of the structures, we performed ab initio molecular dynamics (AIMD) at 300 K using the Nosé–Hoover thermostat within an NVT ensemble.^41,42^ These simulations allowed us to evaluate the stability of the QDs under thermal fluctuations, providing insights into their structural robustness at ambient conditions. For modeling the QDs, we considered two geometrical models, α and β (Figure 1a–d), identified as the most stable structures in ref 33. The primary difference between these QD models lies in the number of dimers bonding the transition metal atoms (*Mo* and *W*) at the edges of the nanotriangles. To systematically explore size-dependent effects, we generated different sizes for each TMD QD by varying *n* from 4 to 10, resulting in seven distinct sizes. We modeled 12 Janus TMD QDs (*MXY*) for each geometric configuration and the pristine phases (*MX*_2_) for comparison. Altogether, 252 nanostructures were studied, categorized into three groups: oxidized Janus QDs, nonoxidized Janus QDs, and pristine QDs (see Table 1).

**Figure 1:**
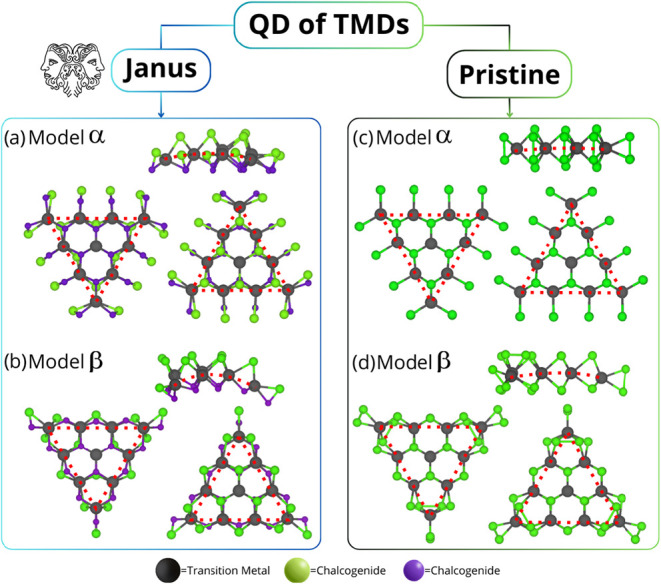
Structural models in ball and stick representation (α and β) used to generate the quantum dots. In the α model, the edge transition metal (*M*) atom is bound to a single *X*–*Y* dimer, whereas in the β model, the edge *M* atom is bonded to two *X*–*Y* dimers. (a) represents the Janus model α, (b) is the Janus model β, (c) depicts the pristine model α, and (d) the pristine model β. The number of transition metal atoms along the nanotriangle edges is denoted as *n* and is highlighted with red dotted lines.

**Table 1:** Simulated QDs, Categorized as Oxidized, Non-Oxidized, and Pristine

oxidized	nonoxidized	pristine
*MoSO*	*MoSSe*	*MoS*_2_
*MoSeO*	*MoSTe*	*MoSe*_2_
*MoTeO*	*MoSeTe*	*MoTe*_2_
*WSO*	*WSSe*	*WS*_2_
*WSeO*	*WSTe*	*WSe*_2_
*WTeO*	*WSeTe*	*WTe*_2_

### 2.1 Formation Energy Formalism

To assess the thermodynamic stability of different sizes and edge terminations of Janus TMD quantum dots (QDs), we employed the formation energy (*FE*) formalism, as defined in eq 1. Our analysis distinguishes between oxidized (*MXO*) and nonoxidized (*MXY*) Janus QDs, using bulk 2*H*–*MX*_2_ structures as a reference to compare the stability of QDs with varying sizes, ranging from *n* = 4 to *n* = 10 transition metal atoms along the edges. The *FE* per atom incorporates the chemical potentials of each atomic species in the general formula *M*_*x*_ *X*_*y*_ *Y*_*z*_.

1 $$\mathrm{FE}=\frac{E_{M_{x}X_{y}Y_{z}}-x\mu_{M}-y\mu_{X}-z\mu_{Y}}{x+y+z}$$

where *E*_*M*_*x*_ *X*_*y*_ *Y*_*z*__ represents the total energy of the Janus TMD QD, while *x*, *y*, and *z* denote the number of each atomic species in the nanotriangle. The chemical potentials of the respective atomic species are denoted as μ_*M*_, μ_*X*_, and μ_*Y*_. For oxidized QDs, the formation energy (*FE*) was computed using eq 1. In contrast, for nonoxidized Janus QDs, the surface formation energy (SFE) was computed relative to the bulk phases of TMDs. Thermal equilibrium among the bulk, vacuum, and molecular structures was considered to establish the following correlation among chemical potentials

2 $$\mu_{M}^{\mathrm{Bulk}}+2\mu_{X}^{\mathrm{Bulk}}+\Delta H_{f}=\mu_{MX_{2}}=\mu_{M}+2\mu_{X}$$

where Δ*H_f_* represents the formation enthalpy of the bulk *MX*_2_ reference phase. The chemical potentials of the *X* species are further constrained by their respective bulk phases

3 $$\mu_{X}\leq \mu_{X}^{\mathrm{Bulk}}$$

since

4 $$\mu_{MX_{2}}^{\mathrm{Bulk}}=\mu_{M}+2\mu_{X}$$

Thus, the surface formation energy (SFE) can be rewritten using eqs 1 and 4 as follows

5 $$\mathrm{SFE}=\frac{E_{M_{x}X_{y}Y_{z}}-x\mu_{MX_{2}}^{\mathrm{Bulk}}+\mu_{X}\left(2x-z\right)-y\mu_{Y}}{x+y+z}$$

Furthermore, for pristine phases, the SFE is calculated using the following expression

6 $$\mathrm{SFE}=\frac{E_{M_{x}X_{y}Y_{z}}-x\mu_{MX_{2}}^{\mathrm{Bulk}}+\mu_{X}\left(2x-z\right)}{x+y}$$

## 3 Results and Discussion

### 3.1 Structure and Curvature Evolution

We performed a complete structural optimization without any constraints in all 252 QDs. Interesting patterns were observed regarding QD size, atomic radius, and electronegativity. Focusing on the pristine QDs, we observed that they remain almost flat for *MoS*_2_ (Figure S1a), *MoSe*_2_ (Figure S1b), and *MoTe*_2_ (Figure S1c) in the α configuration. In contrast, in the β configuration, QDs depict a slight curvature at the nanotriangles’ vertices related to a size effect. This is expected since there is no difference in electronegativity between the upper and lower chalcogen layers. Atomistic models of the relaxed *n* = 10 QDs for the α and β pristine *MoS*_2_, *MoSe*_2_, and *MoTe*_2_ models are shown in Figure 2a.

**Figure 2:**
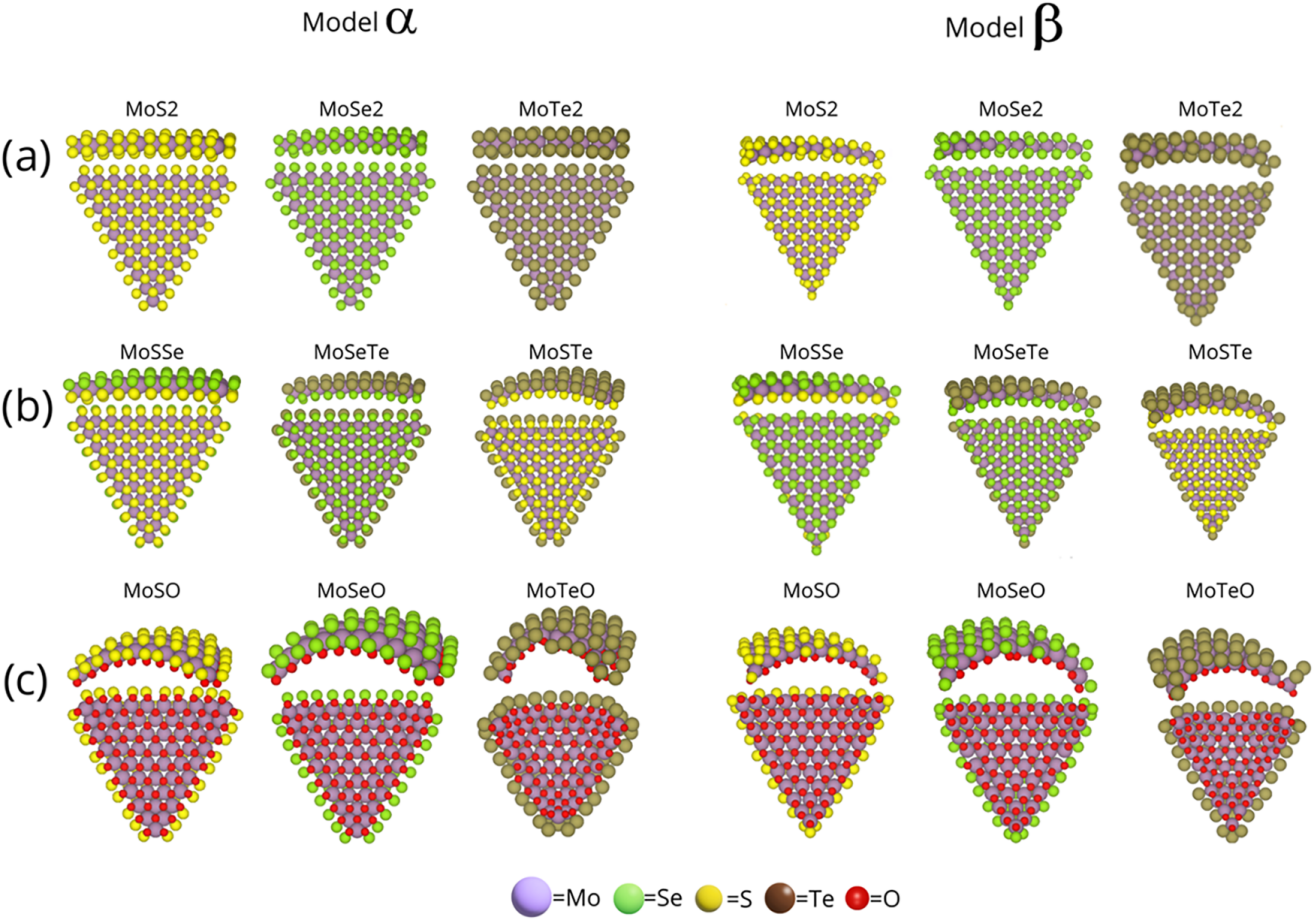
Pristine and Janus quantum dots (QDs) (*n* = 10). (a) Corresponds to pristine α and β models, (b) depicts the Janus nonoxidized Mo-based QDs, and (c) the Janus oxidized Mo-based QDs.

Interesting things appear when analyzing the nonoxidized Janus QDs. First, in this case, the different chalcogen atoms in the upper and lower parts of the QDs generate a charge density asymmetry, which induces a curvature proportional to the electronegativity difference between the chalcogen atoms. Notice in Figure S2a the α and β models for the Janus *MoSSe* QDs, where the slight electronegativity difference in Pauling scale between *S* (2.58) and *Se* (2.55) induces curvature. The curvature is generated toward the layer with the largest electronegativity and lowest atomic radius. Notice here that the nonbonded *S* atomic radius is 1.80 Å and *Se* 1.90 Å (the same trend in size is observed if considering covalent radius). The curvature increases as the difference in electronegativity increases, as seen in Figure S2b, the Janus *MoSeTe* QDs present a curvature larger than the *MoSSe* QDs. Here, the difference in electronegativities between *Se* (2.55) and *Te* (2.10) is larger, by 0.45. Here, we observe a collective effect that includes size, charge density asymmetry, and atomic radius. Once more, notice that the curvature happens toward the most electronegative and small species. The final piece of evidence is generated when studying the *MoSTe* QDs, see Figure S2c. In such a case, the electronegativity difference is 0.48, inducing a curvature larger than in the previous two Janus QDs. The same pattern is repeated, curvature is directed toward the most electronegative and small chalcogen atom, see the *n* = 10 atomistic models for the *MoSSe*, *MoSeTe*, and *MoSTe* in Figure 2b.

Now, when analyzing the oxidized Janus QDs in their α and β configurations, we can observe that these models have a pronounced curvature. Figure S3a depicts the Janus MoSO, where the difference in electronegativity between O and *S* is 0.86. Compared to the previous structures, these have the largest curvature. Now, if we compare Janus *MoSO* with Janus *MoSeO* (Figure S3b) and Janus *MoTeO* (Figure S3c), the electronegativity differences are still larger, being 0.89 and 1.34 for the *O*–Se and *O*–Te atoms, respectively. Notice that the behavior is the same, and the curvature gets pronounced for the oxidized structures. See Figure 2c to compare between models with *n* = 10 where the curvature effect is evident. There is also an effect on the atomic size, curvature increases following a trend in the upper layer content as follows *S* < *Se* < *Te*. The TMDs curvature in terms of electronegativity and atomic size difference follows the order: for pristine QDs *MoS*_2_ < *MoSe*_2_ < *MoTe*_2_, for nonoxidized Janus QDs *MoSSe* < *MoSeTe* < *MoSTe*, and for oxidized Janus QDs *MoSO* < *MoSeO* < *MoTeO* (Figure 2).

A similar trend is observed in the W-based QDs for the α and β configurations. Figure S4 depicts the optimized pristine *WS*_2_, *WSe*_2_ and *WTe*_2_ QDs. Figure S3a shows the size evolution in pristine *WS*_2_, as before, the size increases from *n* = 4 to *n* = 10, depicting a very slight increase in curvature due to QD size. Similar characteristics appear for *WSe*_2_ (Figure S4b) and *WTe*_2_ (Figure S4c). As in the case of Mo-based QDs, interesting things appear when the electronegativity difference breaks the symmetry. Janus *WSSe*, *WSeTe*, and *WSTe* are depicted in Figure S5a–c. The same effect as in the Mo-based QDs is evident, the curvature increases as the difference in electronegativity does, following the order *WSSe* < *WSeTe* < *WSTe* in both α and β models. Finally, when analyzing the oxidized Janus QDs, the trend is also the same, with curvature of both α and β models increasing as follows: *WSO* < *WSeO* < *WTeO*, see Figure S6a–c.

When comparing Mo- and W-based QDs, the curvature increases with the number of transition-metal atoms along the edges, consistent with progressive accumulation of edge-induced strain in larger nanotriangles. For a given composition, W-based systems exhibit systematically larger out-of-plane deformation than their Mo counterparts, likely due to stronger bonding interactions and reduced structural flexibility. Oxidation further amplifies this effect: Pristins *MoX*(2)/*WX*(2) structures remain nearly planar, nonoxidized Janus *MoXY*/*WXY* systems show moderate curvature that increases with chalcogen mismatch, and oxidized *MoXO*/*WXO* exhibit the largest deformation following the trend *S* < *Se* < *Te*. This effect is clearly observed in Figure 3, where the structural distortion is quantified through the Δ*Z* descriptor, defined as the difference in the out-of-plane coordinate between the vertex atoms and the central transition-metal atoms. Larger Δ*Z* values indicate a stronger out-of-plane deformation and, therefore, a more pronounced buckling of the optimized quantum dots.

**Figure 3:**
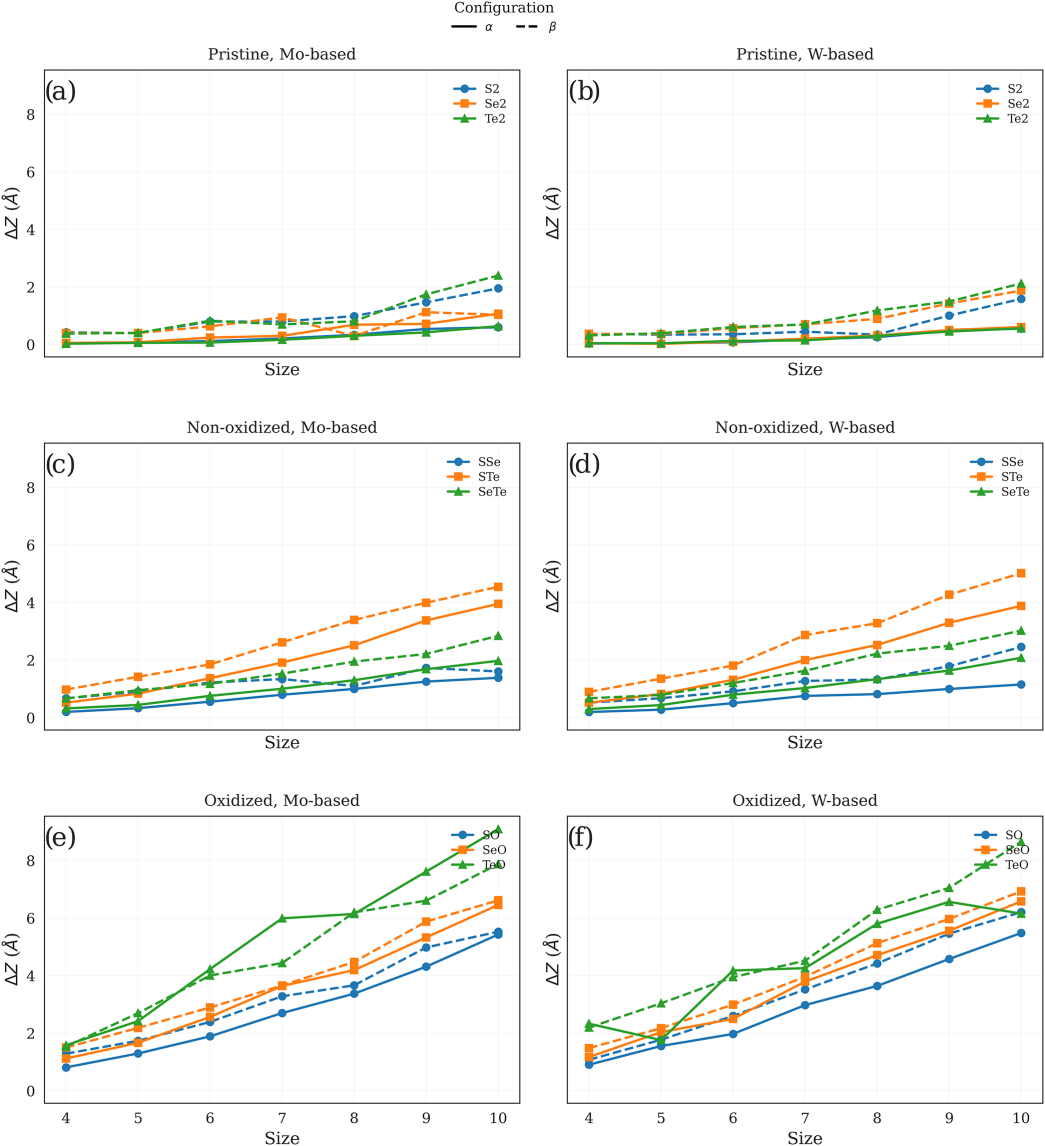
Variation of the out-of-plane distortion descriptor, Δ*Z* = *Z*_max_ – *Z*_min_, as a function of quantum dot size for the α and β configurations of the studied systems. The panels are organized according to the chemical family (pristine (a, b), nonoxidized Janus (c, d), and oxidized (e, f)) and the transition-metal center (Mo-based and W-based). Solid lines correspond to the α structures, whereas dashed lines represent the β structures. This descriptor provides a practical measure of the overall buckling amplitude of the optimized quantum dots.

Previous theoretical studies have reported size-dependent curling regimes in Janus TMD nanostructures, including uniform bowl-like bending in small flakes and more complex deformation patterns in larger domains.^43,44^ The quantum dots treated here (*n* = 4–10) fall within the small-size regime, where bending rigidity is reduced and global bowl-like curvature is energetically favorable. In our analysis, the electronegativity and atomic-size mismatch between chalcogen layers generate asymmetric bonding and residual stress across the two faces of the nanotriangles. This residual stress is consistent with the intrinsic strain reported in previous MD and FEM studies^43,44^ and is relieved through out-of-plane deformation, as quantitatively shown in Figure 3.

### 3.2 Stability Analysis

Figure 4a depicts the formation energies of the *Mo*-based nanotriangular quantum dots vs edge size (*n*), including edge geometry (α and β, see Figure 1). A general stabilization trend is observed as the size increases across all configurations: as *n* increases from 4 to 10, the formation energies become systematically more stable, in line with the previously observed trend for *MoS*2 and *MoSeS* nanotriangles.^45^

**Figure 4:**
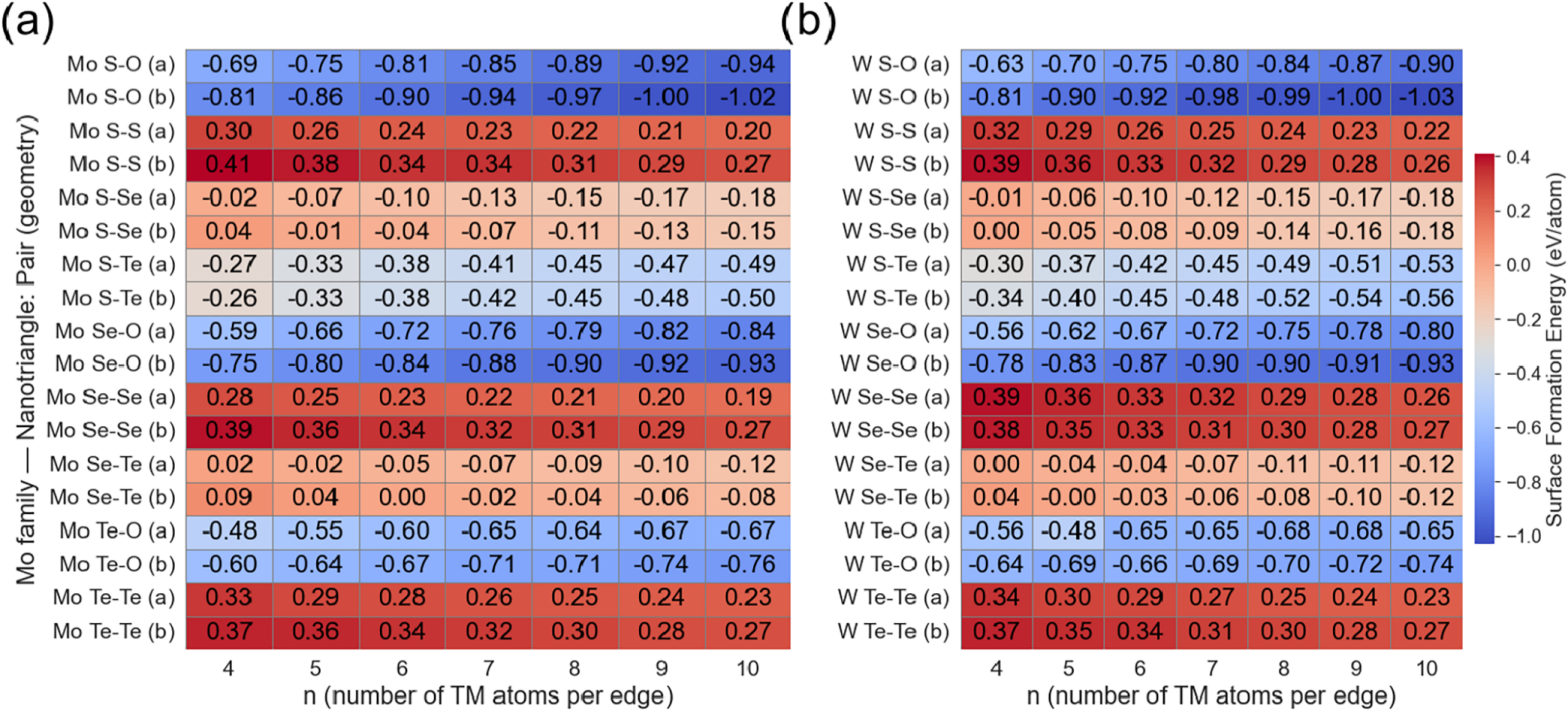
Annotated heatmap summarizing the formation energies vs number of atoms per edge in (a) *MoXO* and (b) *WXO* families. The formation energy (FE) is computed using eq 1.

Focusing on the pristine structures (S–S, Se–Se, and Te–Te), these remain with positive formation energies across the studied range but still show stabilization as the size of the nanotriangles increases. For instance, S–S­(α) formation energy stabilizes from 0.30 eV/atom at *n* = 4 to 0.20 eV/atom at *n* = 10, with the β geometry being consistently less stable. The same trend is observed in the Se–Se and Te–Te structures, with formation energies ranging from 0.28 eV/atom at *n* = 4 to 0.19 eV/atom at *n* = 10, and from 0.33 eV/atom at *n* = 4 to 0.23 eV/atom at *n* = 10, respectively, indicating the same stabilization tendency. As in the S–S nanotriangles, the β model is less stable than the α model for both Se–Se and Te–Te systems.

In sharp contrast, Janus structures induce a pronounced chemical stabilization that depends strongly on the electronegativity of the substituted chalcogen and on the resulting modification of the existing Mo–X bonds. Focusing on S–O, Se–O, and Te–O nanotriangles, these are the most stable of all the series, always displaying negative formation energies even at *n* = 4 and becoming progressively more stable as n increases. As an example, notice that the S–O (β) model formation energy decreases from −0.81 eV/atom at *n* = 4 to −1.02 eV/atom at *n* = 10. This stabilization arises mainly from the strong polarity induced by the difference in electronegativity between S and O, from the enhanced strength of the Mo–O bonds, and from the enhanced strength of the Mo–O bonds. Here, we observe that the stability trend reverses, with the β models being more stable than the α models, and both show negative formation energies across all O-containing nanotriangles. The stability trend in these nanotriangles is as follows: S–O > Se–O > Te–O, indicating that the electronegativity difference alone is not the sole stability descriptor; bond strength and atomic size differences also contribute to stabilization, with smaller chalcogens forming more compact and energetically favorable structures.

Upon analyzing the mixed non-O-containing Janus nanotriangles, an intermediate behavior is observed, characterized by size-dependent stabilization. Systems as S–Te are stable throughout the entire size range, while S–Se and Se–Te display a transition from positive to negative formation energies at critical sizes. S–Se (α) stabilizes at *n* = 4, and the β model at *n* = 5, while the Se–Te (α) nanotriangles become stable at *n* = 5, and the corresponding β geometry reaches negative formation energies at larger size (*n* = 7). These trends indicate that, for non-O Janus nanotriangles, stabilization is governed by a combined effect of electronegativity differences, atomic-size mismatch, and bond strength. Accordingly, the stability order follows S–Te > S–Se > Se–Te.

With respect to the W-based nanotriangle quantum dots, Figure 4b depicts the formation energies vs edge size. As in the Mo-based models, all the W-based quantum dots exhibit systematic stabilization upon increasing size. Here, the formation energies remain strongly dependent on the chemical nature of the nanotriangles, with the O-containing structures being the most stable, followed by the Janus non-O containing, and the least stable are the pristine nanotriangles.

Focusing on the S–S, Se–Se, and Te–Te structures, they hold positive formation energies across the entire size range. It is interesting to notice that these structures stabilize monotonically with increasing *n*. This trend is the same as one seen in the Mo-based quantum dots. In the S–S (α) system, the formation energy goes from 0.32 eV/atom at *n* = 4 to 0.22 eV/atom for *n* = 10, while in the β nanotriangles, the stabilization goes from 0.39 eV/atom for *n* = 4 to 0.26 eV/atom for *n* = 10. The same behavior is observed in the Se–Se and Te–Te (α) structures, with a stabilization of around 0.1 eV/atom from *n* = 4 to *n* = 10. Also, notice that α models are more stable than β models for all nanotriangle sizes. The O-containing Janus W-based nanotriangles S–O, Se–O, and Te–O exhibit negative formation energies even at small sizes and follow the same trend of increasing stability with increasing size. It is also worth noting that β models are more stable than α models. As in the case of Mo nanotriangles, the stability is mainly conferred by the formation of strong W–O bonds and by the intrinsic Janus-induced electronic asymmetry between chalcogen layers. The stability trend follows S–O > Se–O > Te–O, consistent with that observed for Mo-based quantum dots.

Finally, the non-O Janus W-based nanotriangles exhibit intermediate stability and the same stability-size dependence. Notice that the S–Te system is stable across the whole size range, whereas S–Se and Se–Te exhibit transitions from positive to negative formation energies. Here, the α models are more stable than the β models. The α model of S–Se nanotriangles is stable at all sizes, whereas in the β models, the transition from positive to negative formation energy occurs at *n* = 5. Stability shifts to larger nanotriangles in the Se–Te models. For the α nanotriangles, the transition from positive to negative formation energy happens at *n* = 5, and for β at *n* = 7. These trends reflect the combined influence of atomic-size mismatch and electronegativity differences on the stabilization of non-O Janus nanoparticles, leading to the stability order S–Te > S–Se > Se–Te.

Although fully ordered MoSO or WSO Janus quantum dots have not yet been experimentally reported, oxygen incorporation into MoS2 lattice has been already demonstrated under oxidative and catalytic conditions.^46^ Spectroscopic evidence confirms the formation of stable Mo–O bonds and the partial substitution at sulfur sites, while preserving the layered structure. In this context, the MoXO and WXO configurations studied here may be regarded as oxygen-functionalized Janus derivatives in the limiting-substitution case. The formation energy analysis discussed earlier showed that oxygen incorporation increased the thermodynamic stabilization of the O-containing Janus structures, possibly guiding further experimental efforts to control oxidation, as in ref 46, or to develop postgrowth modification strategies.

#### 3.2.1 Thermal Stability of *MoSO*MoSO and *WSO*WSO Quantum Dots

To assess the thermal stability of MoSO and WSO nanotriangles, ab initio molecular dynamics (AIMD) simulations were performed at 300 K for five ps. Both α (Figure 5) and β (Figure 6) models were considered in two sizes, *n* = 4 (Figures 5a and 6a) and *n* = 10 (Figures 5b and 6b). For MoSO quantum dots, the AIMD trajectories indicate that both geometries remain structurally intact at room temperature. Notice that the energy equilibrates rapidly within the first ∼1.0 ps and subsequently remains around a well-defined mean value. The α model (Figure 5) exhibits slightly larger energy and temperature fluctuations than the β model (Figure 6), indicating higher sensitivity to thermal excitations. This behavior is governed by the combined effects of edge structure and the local chemical environment. The presence of oxygen strengthens and polarizes the metal-chalcogen bonds, increasing the stiffness of the bonding network and promoting a more coherent structural response. The β geometry (Figure 6b) in O-containing nanotriangles provides an edge motif that more effectively accommodates this chemically strengthened and polarized bonding environment by distributing strain and Janus-induced polarization across a large number of edge bonds, resulting in reduced atomic mobility and smoother energy trajectories. In contrast, the α geometry (Figure 5b) concentrates the same asymmetry into a sharper edge motif, which favors larger local relaxations and, in turn, larger thermal fluctuations. It is important to note that in either case, there is no bond breaking, reconstructions, or loss of triangular morphology, indicating that both models are thermally stable on the AIMD time scale, consistent with their negative formation energies.

**Figure 5:**
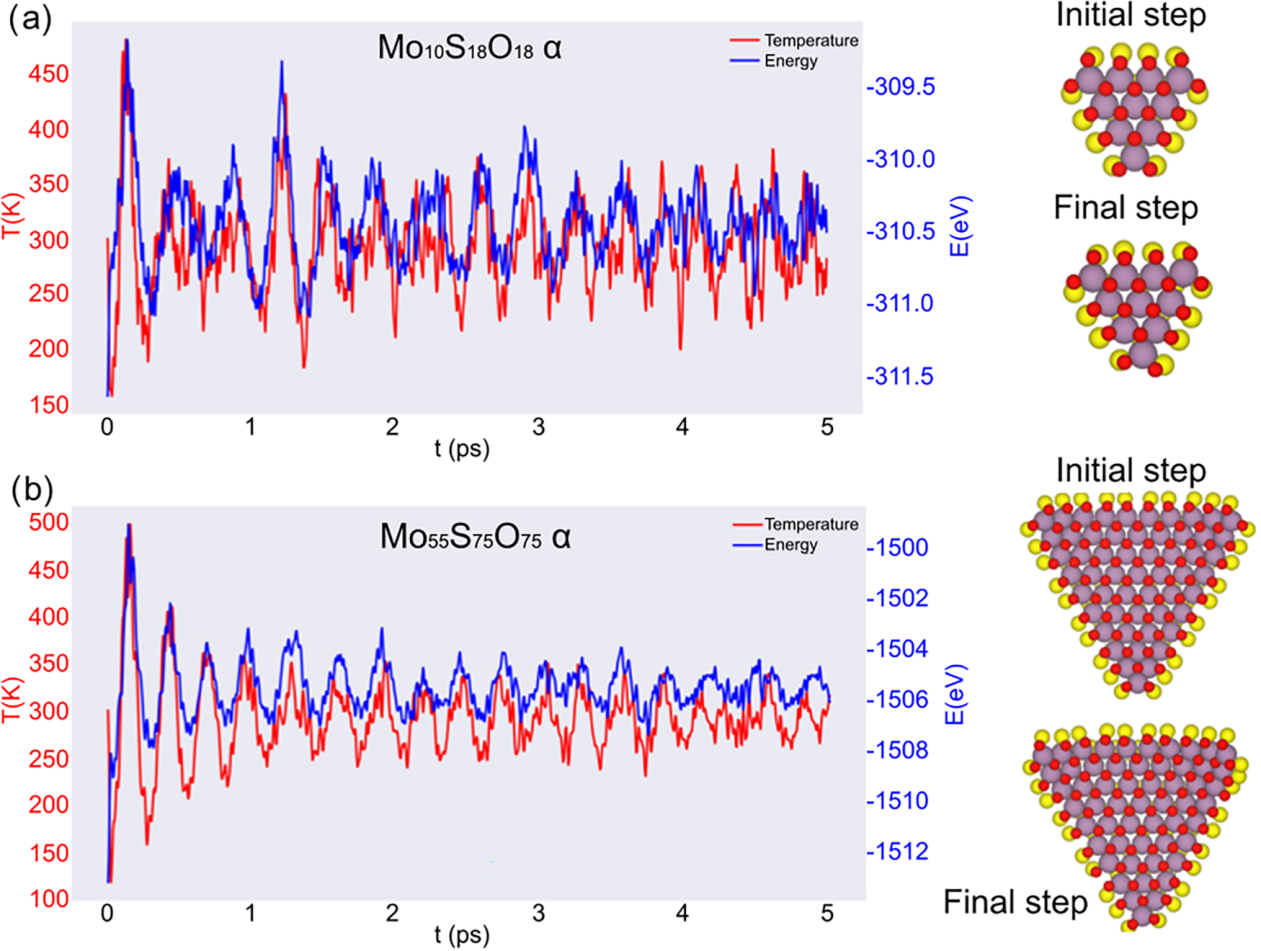
Atomistic structures and AIMD simulations at 300 K of *MoSO* nanotriangles in the α configuration: (a) *n* = 4 and (b) *n* = 10.

**Figure 6:**
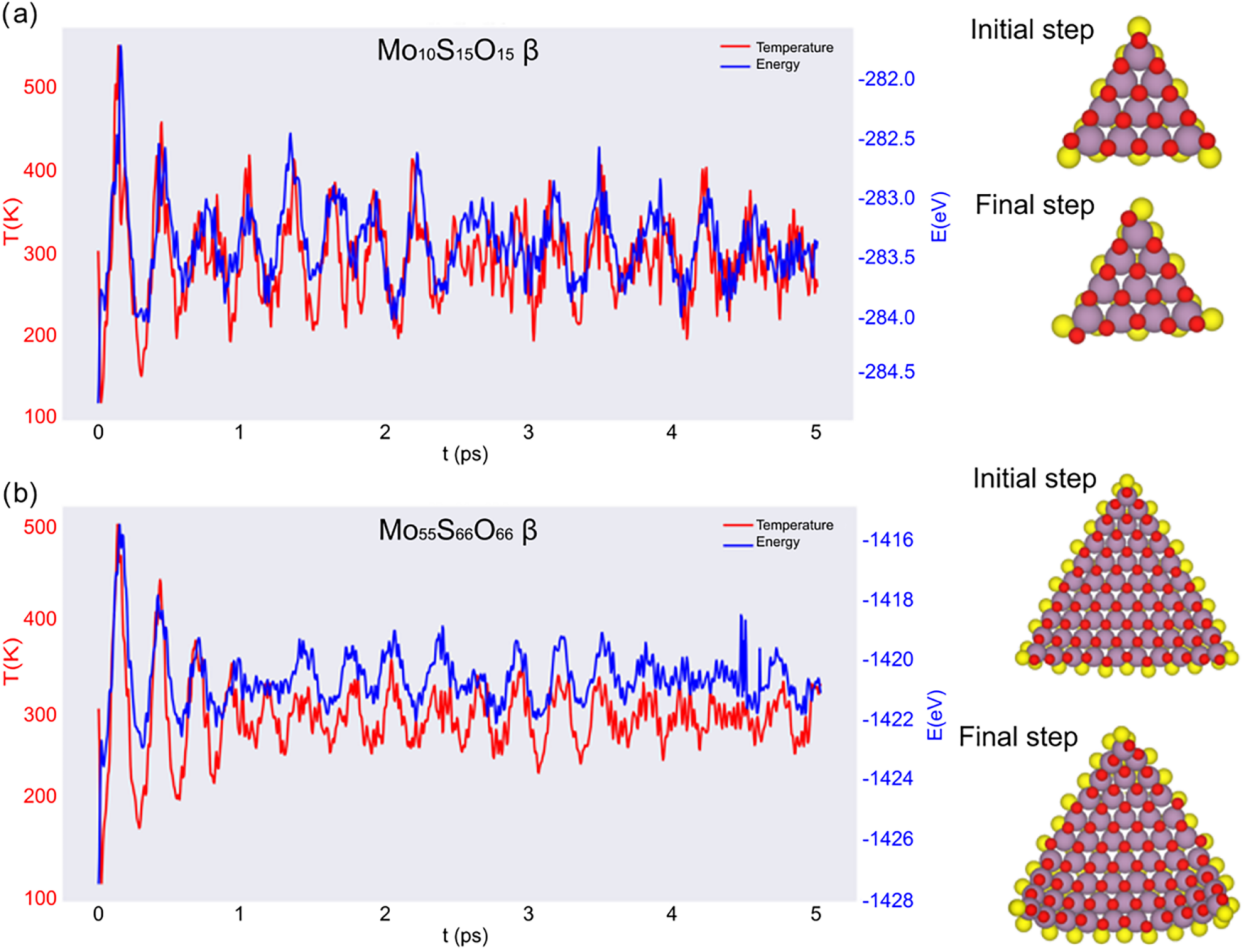
Atomistic structure and AIMD at 300 K of the *MoSO* nanotriangles in β configuration for: (a) *n* = 4 and (b) *n* = 10.

A similar behavior is observed for WSO nanotriangles, with a pronounced size dependence. The smaller WSO nanotriangles (*n* = 4) displays relatively large temperature and energy fluctuations during the early stages of the simulation (Figures S7a and S8a), reflecting higher atomic mobility. Still, the system stabilizes as the simulation progresses, and the final configuration closely resembles the initial one. For larger nanotriangles (*n* = 10) (Figures S7b and S8b), the energy fluctuations are overall smoother and more regular, particularly for the α model (Figure S7b). The β geometry (Figure S8b) remains dynamically stable but exhibits persistent oscillatory fluctuations throughout the simulation. As in Mo-based systems, this enhanced stability arises from the combined effects of increased size and the presence of strong, polar W–O bonds, which chemically reinforce the lattice and reduce the sensitivity of both edge and interior atoms to thermal perturbations. This dynamical behavior directly mirrors the enhanced thermodynamic stabilization identified from the formation energy analysis.

Upon comparing all the systems, a consistent size-dependent stability trend emerges. Smaller nanotriangles (*n* = 4) exhibit larger fluctuations in both energy and temperature, reflecting significant atomic mobility and an enhanced contribution of the edge atoms. Despite the fluctuations in the smallest systems, none of the nanotriangles show evidence of bond breaking, structural reconstruction, or loss of triangular morphology in the analyzed time scale. In contrast, larger nanotriangles (*n* = 10) exhibit smoother energy trajectories and lower fluctuation amplitudes, indicating a more rigid bonding network and greater resistance to thermal fluctuations. The persistence of triangular geometry in both α and β configurations confirms that Janus MoSO and WSO nanotriangles are thermally robust at room temperature, in agreement with their thermodynamic stabilization as inferred from formation energies.

### 3.3 Electrostatic Potential Isosurface Analysis

In this subsection, we present a detailed view of the electrostatic potential isosurfaces (EPI) for the O-containing Janus nanotriangles (Figure 7). The colors represent electron-rich regions (red) and electron-depleted regions (blue), highlighting polarity, changes in local chemical environment (surface reactivity), and curvature caused by the asymmetry introduced by the oxygen atoms.

**Figure 7:**
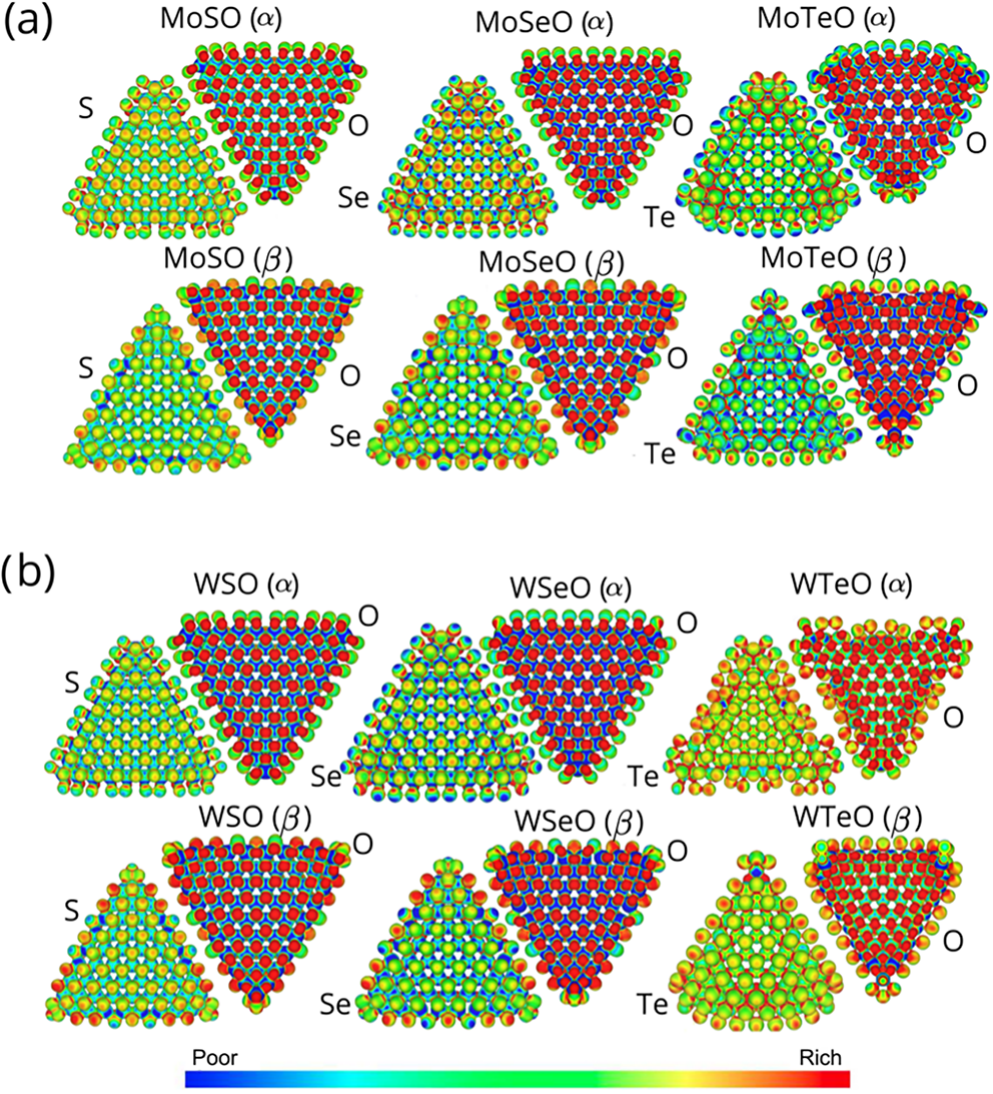
Electrostatic potential isosurfaces for the oxygen-containing Janus nanotriangles: (a) Mo-based and (b) W-based structures. The electrostatic potential is represented by the color scale shown in the bottom panel, where blue corresponds to electron-deficient (low electron density) regions and red corresponds to electron-rich (high electron density) regions; cyan, green, and yellow indicate intermediate values. These potential maps reveal the heterogeneous surface charge distribution associated with the Janus character of the nanotriangles and the presence of oxygen atoms.

In Figure 7a, the left panel shows the EPI for the MoSO (α) model, the electrostatic potential is strongly asymmetric across the nanotriangle. The chalcogen-terminated side exhibits enhanced charge localization along the edges and vertices, whereas the O-terminated face shows a more delocalized potential distributed over the basal plane and edges. This imbalance indicates that the reactivity on the chalcogen side is preferentially directed along the edges, while the oxygen side stabilizes the structure through a more uniform electrostatic landscape. Moving from MoSO to MoSeO and MoTeO (α), the EPI maps reveal a progressive increase in lateral polarization, with charge accumulation becoming more concentrated from left to right. This trend reflects the increasing electronegativity mismatch and the atomic size of the chalcogen species, which enhance curvature and amplify the out-of-plane dipole across nanotriangles.

A similar qualitative behavior is observed in the β configurations (lower panels of Figure 7a), although with notable differences. In these structures, the electrostatic potential on the chalcogen side is more strongly localized at the edges than in the α counterparts, indicating enhanced edge reactivity. In contrast, the oxygen side maintains a relatively uniform potential distribution across the nanotriangle edges and body. This coexistence of edge-localized electrostatic activity and body-centered stabilization promotes curvature and reinforces the formation of a built-in dipole. Notably, the effect is more pronounced for Te-containing systems, where a denser atomic packing suggests stronger curvature and larger electrostatic potential gradient across the structure.

When focusing on the W-based nanotriangles shown in Figure 7b, these follow the same overarching trends but with systematically stronger electrostatic contrast. For WSO, WSeO, and WTeO, the EPI maps reveal sharper potential gradients, particularly along the chalcogen edges, reflecting the expected stronger spin–orbit coupling and more localized d orbitals of W compared to Mo. In both α and β configurations, the oxygen-terminated faces remain comparatively uniform, while the chalcogen sides host localized electrostatic hot spots. This asymmetry reinforces curvature and stabilizes a sizable dipole moment, which is expected to be stronger in W-based systems than in their Mo counterparts.

Figure S9 depicts the nonoxygen-containing nanotriangle EPIs. In these structures, the asymmetry arises exclusively from the chalcogen-chalcogen Janus pairing (S/Se, S/Te, and Se/Te), but without the strong electronegativity contrast generated by oxygen. As a result, the EPI of the α (Figure S9a) and β (Figure S9b) nanotriangles remains asymmetric but significantly more balanced and delocalized charge than the nanotriangles with oxygen on one side. For the Mo-based systems (Figure S9a), the electrostatic potential on the heavier chalcogen side (Se or Te) tends to be more diffuse and localized along the edges, while the lighter chalcogen side (S or Se) exhibits a smoother, more localized potential distribution extending toward the interior of the nanotriangles. This behavior reflects the gradual increase in atomic size and polarizability from S to Te, indicating that the most active nanotriangles are MoSTe, followed by MoSSe, and finally MoSeTe. α and β nanotriangles in Figure S9a,b show a similar behavior, with some activity zones appearing in the Se side of the MoSSe α and β nanotriangles. When focusing on the W-based nanotriangles (Figure S9b). The electrostatic potential asymmetry between S, Se, and Te faces is slightly more pronounced than in the Mo analogues, consistent with the stronger d-orbital localization and larger relativistic effects of W. Nevertheless, the EPI maps still reveal distributed charge across both lighter S and Se faces. Even in the WseTe and WseTe nanotriangles, where the curvature is evident, the electrostatic potential remains smooth across the basal light chalcogen plane, indicating a built-in dipole but lower than in the case of oxygen, where the electronegativity difference is larger.

These electrostatic features suggest potential applicability in photocatalysis, especially those containing oxygen, where strong dipoles and internal fields are crucial indicators of efficient charge separation and directional carrier transport. More studies are still needed to determine the valence-band maximum and conduction-band minimum of the different Janus nanotriangles, as well as their HOMO and LUMO energies, to complete the set of descriptors that define a potential photocatalyst.

## 4 Conclusions

In this work, we have systematically investigated pristine and Janus Mo- and W-based nanotriangular quantum dots, elucidating how size, edge geometry, and chalcogen electronegativity govern their stability, electrostatic response, and thermal robustness. Our results show that the oxygen-containing Janus nanotriangles have the lowest formation energy, followed by the nonoxygen Janus counterparts, and finally by the pristine structures. The β nanotriangles are consistently favored due to their ability to accommodate stronger and more polarized metal–oxygen bonding. Ab-initio molecular dynamics simulations confirmed that both oxygen-containing counterparts remain structurally intact at room temperature, with the larger systems and β configurations exhibiting reduced thermal fluctuations and enhanced rigidity. Electrostatic potential isosurfaces also reveal that oxygen functionalization causes the most noticeable asymmetry, curvature, and internal polarization, with charge localization concentrated around the chalcogen edges and more uniformly toward the oxygen face. These effects become more significant as the electronegativity difference between O and the other chalcogens increases and are more pronounced in W-based systems. In contrast, for the nonoxygen Janus nanotriangles, the electrostatic asymmetry is weaker and more delocalized, caused by a smaller electronegativity difference. The results discussed here establish that oxygen incorporation, edge geometry, and chalcogen identity are key descriptors controlling the stability and internal electrostatic fields of the Janus transition metal dichalcogenide nanotriangles, serving as the basis for rational design in applications where internal polarization and directional charge separation are desirable, as in photocatalysis.

## Supplementary Material



## Notes

Structural models of pristine, Janus nonoxygen-containing, and oxygen-containing Mo- and W-based nanotriangles for sizes *n* = 4 to *n* = 10; AIMD simulations at 300 K for selected systems and configurations; electrostatic potential isosurfaces for Janus Mo- and W-based nanotriangles (S1–S9) (PDF)
