# Supplementary material for: Oxygen-Driven Stabilization and Electrostatic Asymmetry in Janus Mo- and W‑Based Transition Metal Dichalcogenide Nanotriangular Quantum Dots
Source: ACS Omega. 2026 Apr 30;11(19):28539–49. doi: 10.1021/acsomega.6c00678 (PMC13191685; doi:10.1021/acsomega.6c00678)
Supplement: Supplementary file 1 [file ao6c00678_si_001.pdf]

Supporting Information

Oxygen-driven stabilization and electrostatic asymmetry in Janus Mo- and W-based transition metal dichalcogenide nanotriangular quantum dots

Jair Othoniel Dominguez Godinez[a], Raul Eduardo Santoy Flores[b], José Israel Paez Ornelas[c], Rodrigo Ponce Pérez[b], Luis Pellegrin[c], Do Minh Hoat[d,e], Jonathan Guerrero Sánchez[*,b].

a Centro de Investigación Científica y de Educación Superior de Ensenada, Ensenada Baja California, 22860, México.
b Universidad Nacional Autónoma de México, Centro de Nanociencias y Nanotecnología , Ensenada Baja California, 22860, México.
c Universidad Autónoma de Baja California, Facultad de Ciencias, Ensenada Baja California, 22860, México.
d Duy Tan University, Institute of Theoretical and Applied Research, Ha Noi 100000, Vietnam.
e Duy Tan University, School of Natural Sciences, Da Nang 550000, Vietnam.

Email: guerrero@ens.cnyn.unam.mx

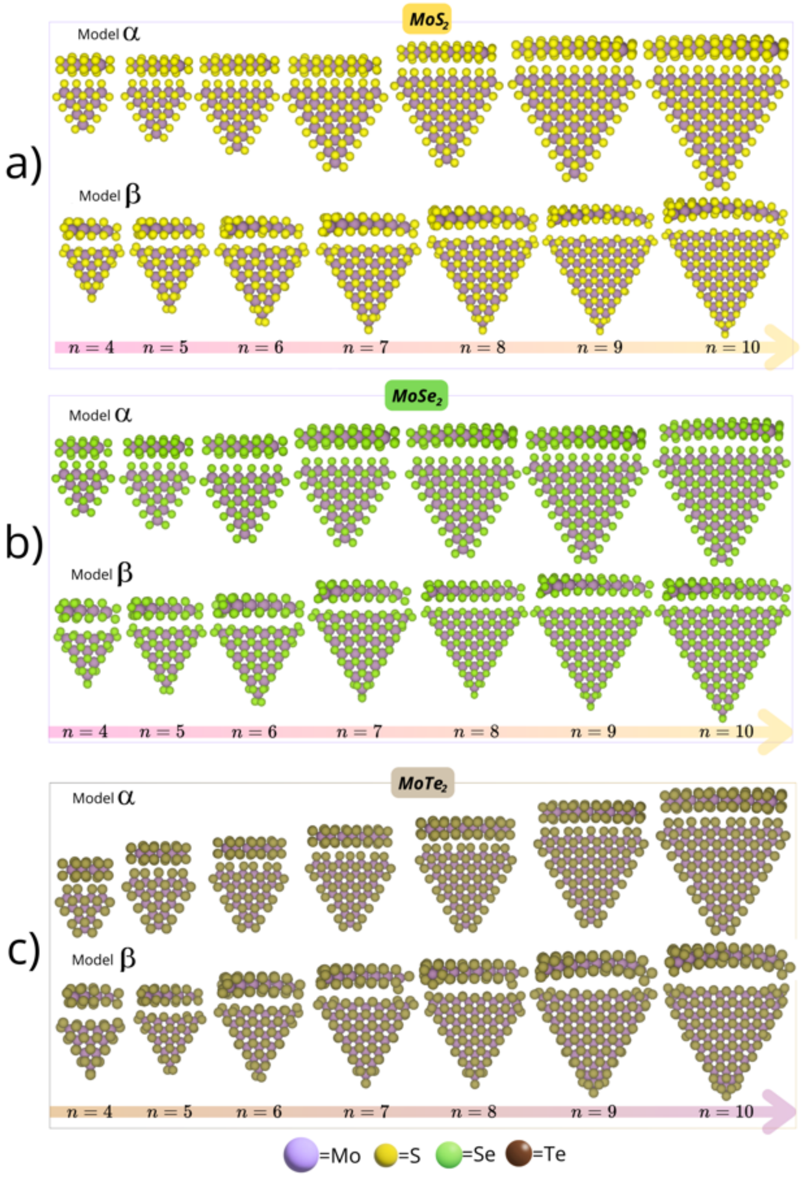

Figure S1: Pristine nanotriangles of different sizes, from $n = 4$ to $n = 10$. (a) MoS_2 in models α and β, (b) $MoSe_2$ in models α and β, and (c) $MoTe_2$ in models α and β.

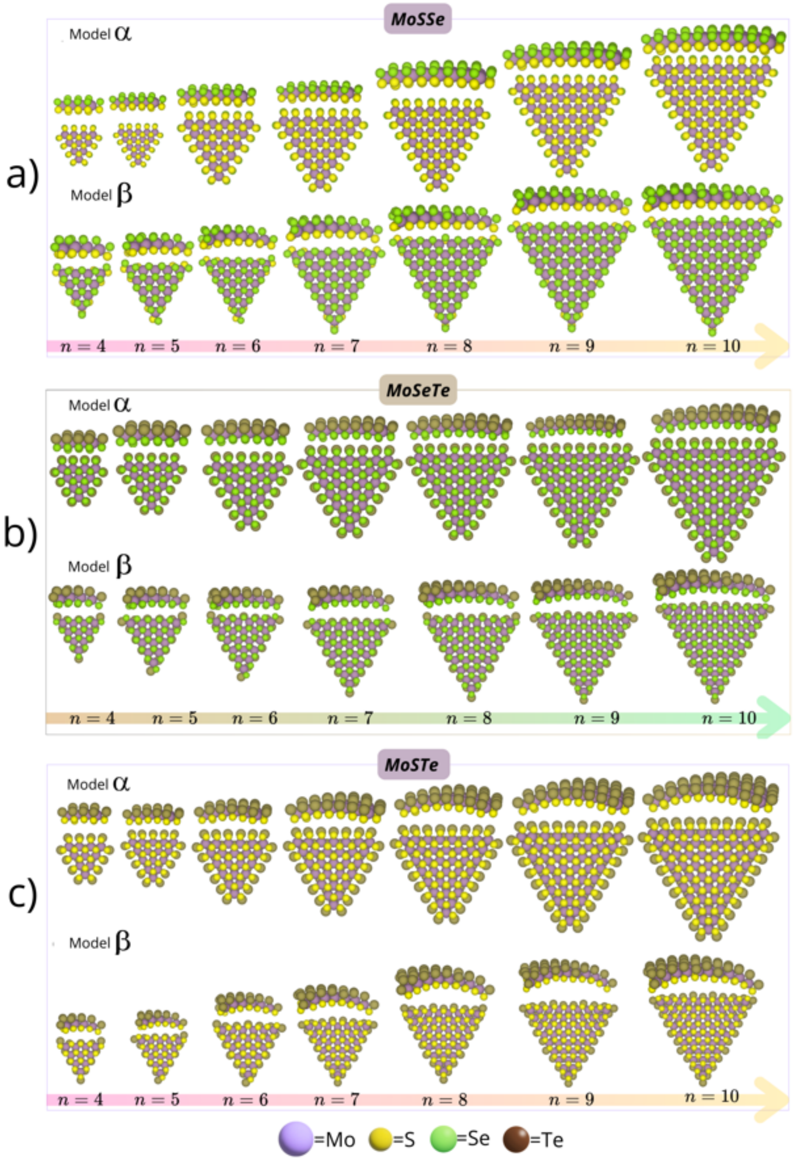

Figure S2: Janus non-O containing nanotriangles of different sizes, from $n = 4$ to $n = 10$. (a) MoSSe in models α and β, (b) MoSeTe in models α and β, and (c) MoSTe in models α and β.

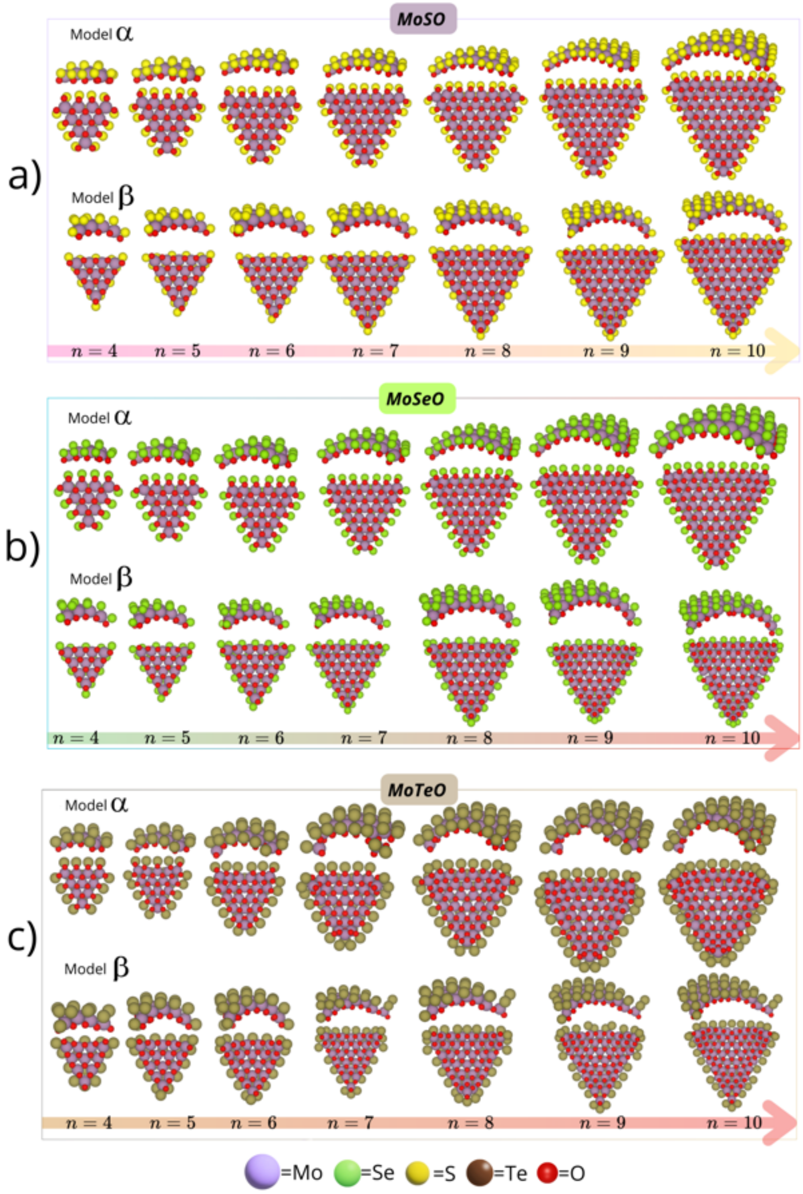

Figure S3: Oxygen containing Janus nanotriangles of different sizes, from $n = 4$ to $n = 10$. (a) MoSO in models α and β, (b) MoSeO in models α and β, and (c) MoTeO in models α and β.

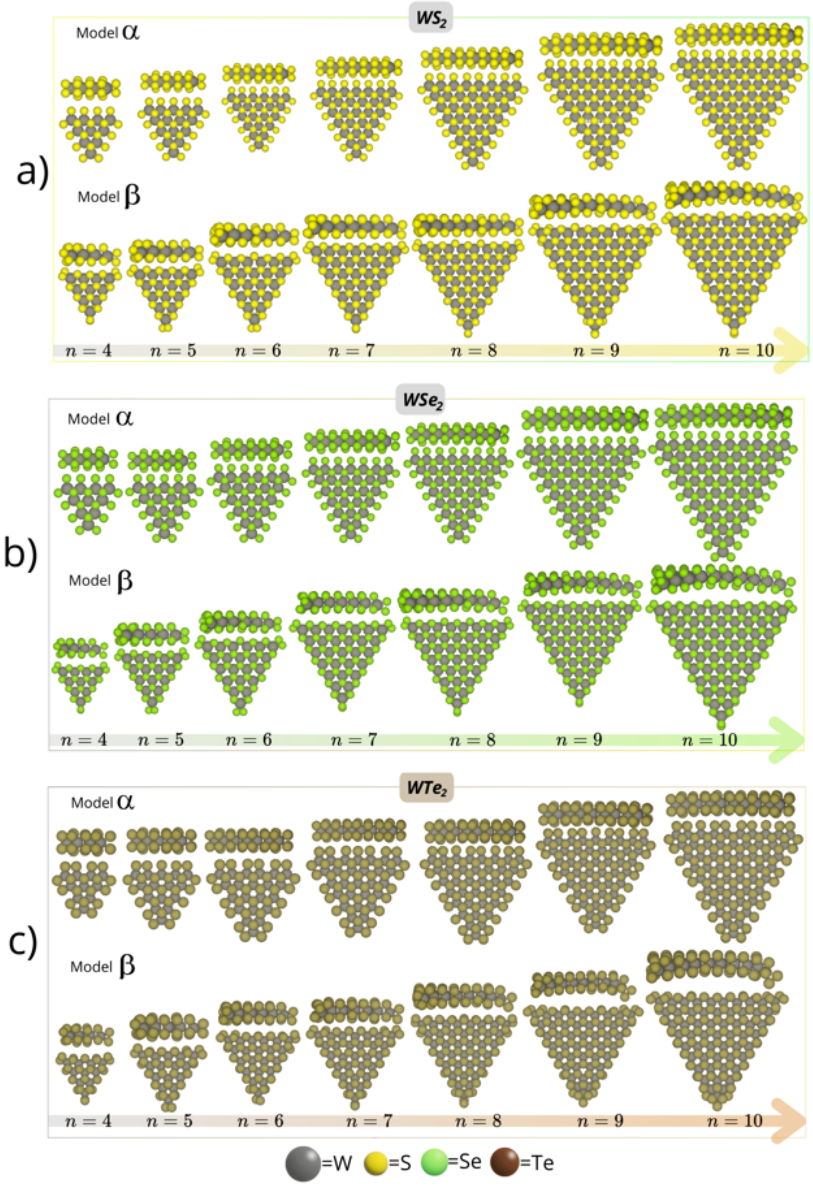

Figure S4: Pristine nanotriangles of different sizes, from $n = 4$ to $n = 10$. (a) WS_2 in models α and β, (b) WSe_2 in models α and β, and (c) WTe_2 in models α and β.

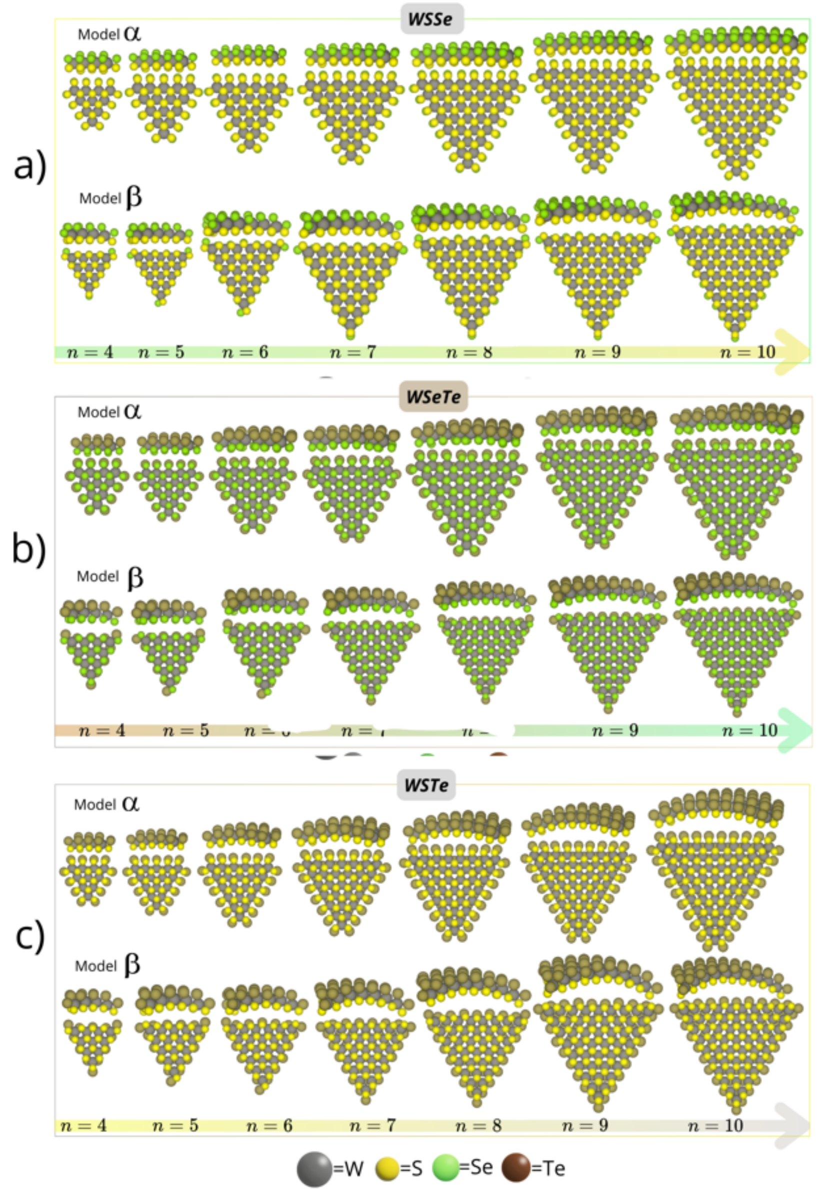

Figure S5: Janus non-O containing nanotriangles of different sizes, from $n = 4$ to $n = 10$. (a) WSSe in models α and β, (b) WSeTe in models α and β, and (c) WSTe in models α and β.

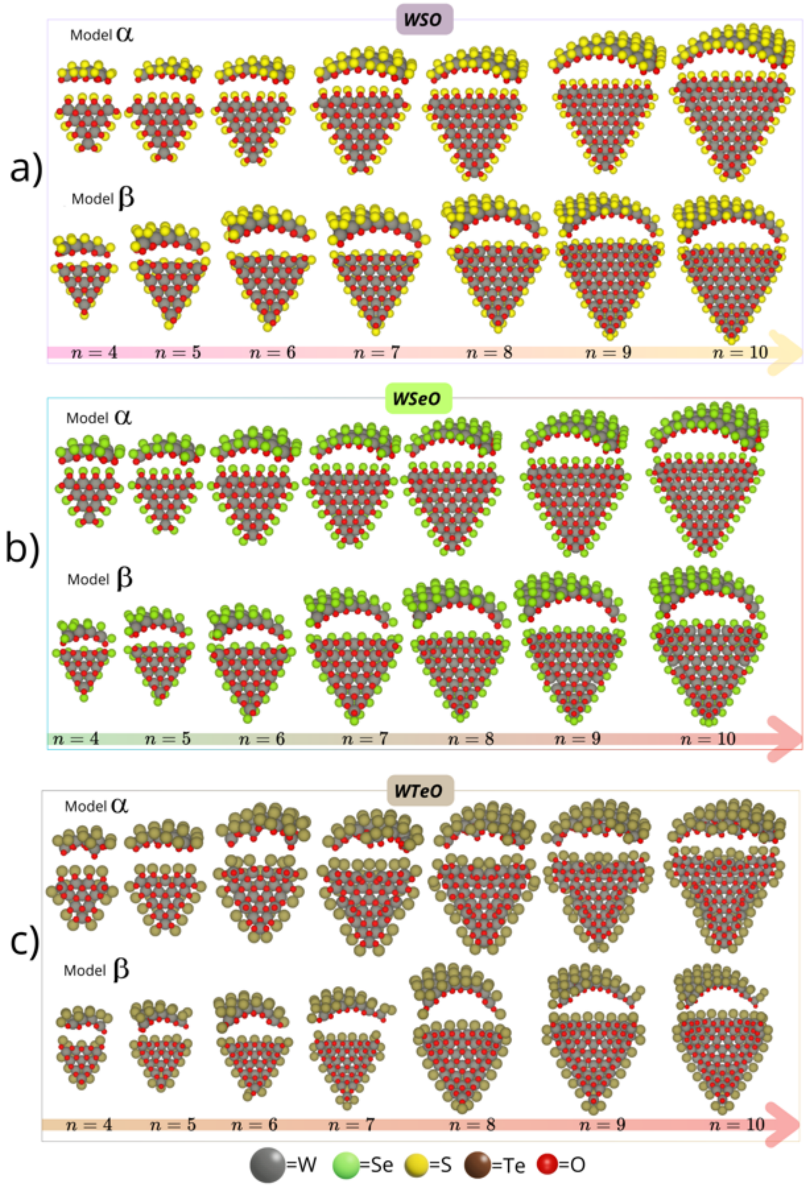

Figure S6: Oxygen containing Janus nanotriangles of different sizes, from $n = 4$ to $n = 10$. (a) WSO in models α and β, (b) WSeO in models α and β, and (c) WTeO in models α and β.

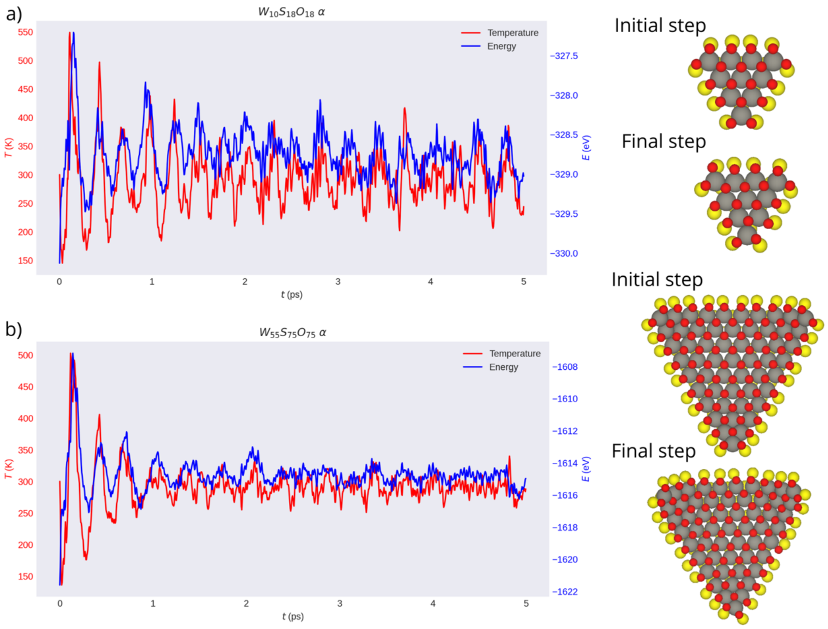

Figure S7: Atomistic structure and AIMD at 300 K of the WSO nanotriangles in α configuration for (a) $n = 4$ and (b) $n = 10$.

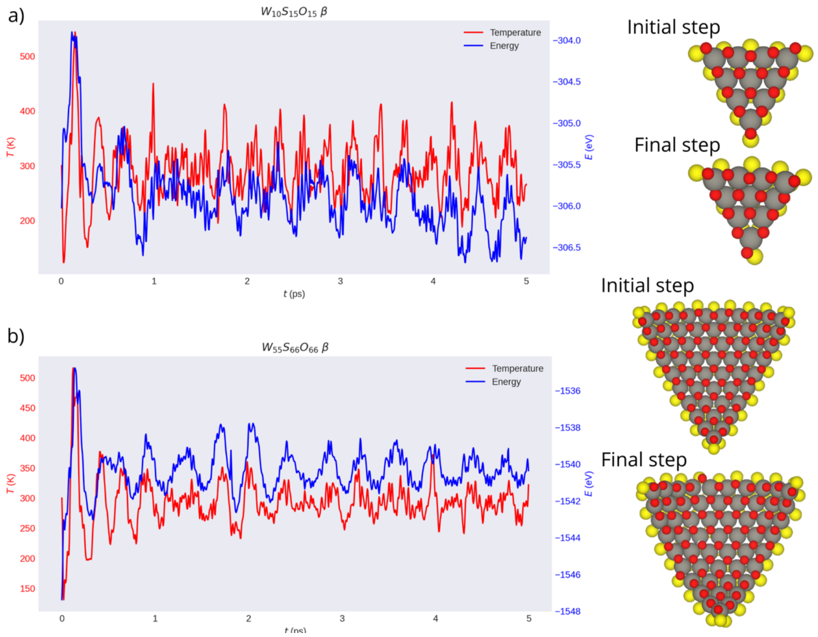

Figure S8: Atomistic structure and AIMD at 300 K of the WSO nanotriangles in β configuration for (a) $n = 4$ and (b) $n = 10$.

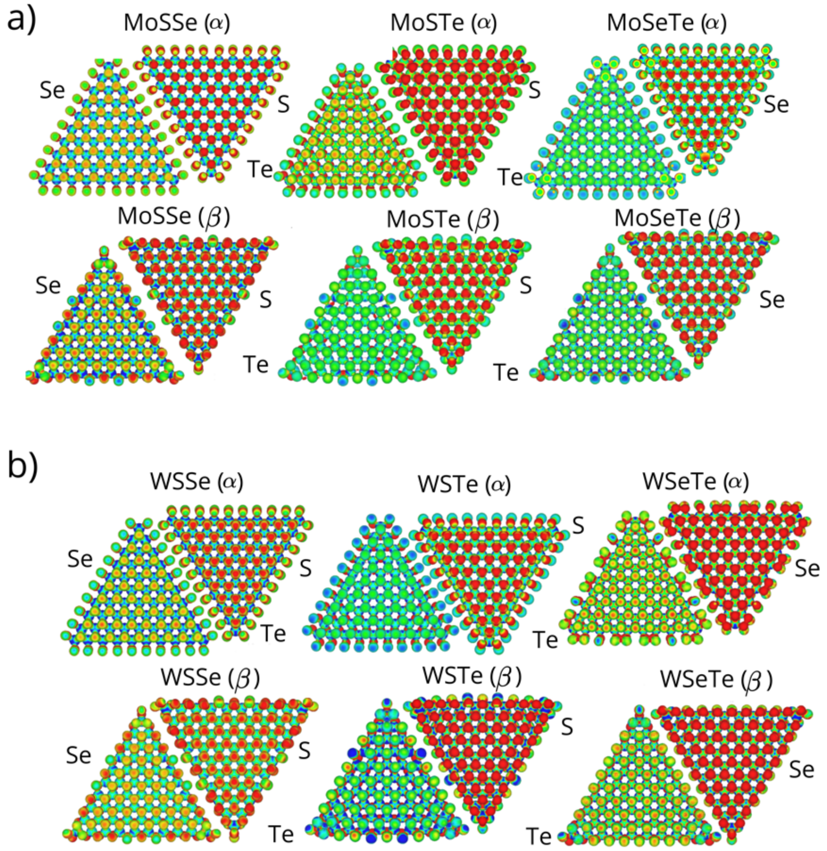

Figure S9: Electrostatic potential isosurfaces of the non-oxygen containing (a) Janus Mo-based nanotriangles, and (b) W-based nanotriangles.
